# Bio-hydrogen production by co-digestion of domestic wastewater and biodiesel industry effluent

**DOI:** 10.1371/journal.pone.0199059

**Published:** 2018-07-11

**Authors:** Jyotsana Prakash, Rakesh Sharma, Sanjay K. S. Patel, In-Won Kim, Vipin Chandra Kalia

**Affiliations:** 1 Department of Chemical Engineering, Konkuk University, Seoul, Republic of Korea; 2 CSIR–Institute of Genomics and Integrative Biology (IGIB), Delhi University Campus, Delhi, India; 3 Academy of Scientific & Innovative Research (AcSIR), Anusandhan Bhawan, New Delhi, India; Maharshi Dayanand University, INDIA

## Abstract

The increasing water crisis makes fresh water a valuable resource, which must be used wisely. However, with growing population and inefficient waste treatment systems, the amount of wastewater dispelled in rivers is increasing abominably. Utilizing this freely available waste-water along with biodiesel industry waste- crude glycerol for bio-hydrogen production is being reported here. The bacterial cultures of *Bacillus thuringiensis* strain EGU45 and *Bacillus amyloliquefaciens* strain CD16 produced2.4–3.0 L H_2_/day/L feed during a 60 days continuous culture system at hydraulic retention time of 2 days. An average H_2_ yield of 100–120 L/L CG was reported by the two strains. Recycling of the effluent by up to 25% resulted in up to 94% H_2_ production compared to control.

## Introduction

Availability of clean water is a worldwide crisis. Despite our earth surface being covered with 70% of water, only 2% is a freshwater, of which 3/4th is frozen and unavailable for human consumption [1, 2]. Thus, billions of people live with severe water scarcity and poor sanitation. The small amount of available fresh water faces an allocation and competition in agricultural, industrial and municipal sectors. As a result, allocating this sparsely available fresh water to bioenergy production is a very costly affair [3]. During bioenergy production, the substrate occupies 10% of the medium while the rest is water. This water used in most of the studies is distilled and the medium is sterilized [4–6]. Since most of the population struggles for fresh water for their daily basic needs, it would be unethical to divert it towards increasing energy demands. The possible solution would be to use wastewater that is generated from domestic and industrial sources. As per the 2016 report published by International Institute of Health and Hygiene, in metro cities like New Delhi (India) about 6.1×10^4^ million liters (ML) of wastewater is generated every day. The treatment capacity is around 50% only (**http://www.sulabhenvis.nic.in/Database/STST_wastewater_2090.aspx**). The rest of the wastewater is drained into the rivers or can meet a less dreadful fate if utilized e.g., for bioenergy production. The surplus availability of wastewater makes it a timeless resource for the researchers struggling with cheap and steady bioenergy generation [7].

Bioenergy being a sustainable alternative to fossil fuels has attracted a huge worldwide support. Hydrogen (H_2_), Methane (CH_4_), ethanol, bio-diesel are amongst the most widely studied bio-fuels. H_2_ however has gained immense favors owing to its high calorific value and cleaner combustion [8, 9]. Most extensively studied technique for biological H_2_ production is dark fermentation and is most likely to be commercialized in near future [10]. A variety of organic wastes have been used successfully as substrate for H_2_ production. This substrate is mostly present along with some minerals in distilled water [11]. The challenge is thus to replace the valuable clean water with readily available domestic wastewater for H_2_ production. H_2_ production from various industrial wastewaters such as cassava starch processing wastewater, brown sugar wastewater, paperboard mill wastewater, ethanol wastewater, etc. have been reported [12–18]. Sugar rich wastewaters such as molasses wastewater, sugarbeet wastewater and sugarcane vinasse have the ability to produce high H_2_ yields of around 3.2 mol/mol substrate. Starchy wastewaters generally result in relatively lower H_2_ yields of around 1.9 mol/mol substrate [17, 19, 20]. Wastewaters from biodiesel industry, which are rich in glycerol also have a potential to produce bioenergy [21, 22]. Use of crude glycerol (CG) as feed prepared in distilled water resulted in 165 L H_2_/L CG by an immobilized biofilm forming bacteria *B*. *amyloliquefaciens* [23]. The use of different industrial wastewaters as medium may not be available throughout the year and may thus hinder the continuity of bio-H_2_ production. In contrast, domestic wastewater which is generated everyday throughout the globe may be a better option to counter this problem. Therefore, in the present study we have used freely available domestic wastewater as the medium and biodiesel industry waste- CG as the substrate for an economic bioenergy generation. To further improve the production efficiency, recycling of the effluent is also reported.

## Material and methods

### Organisms and growth conditions

#### Hydrogen producers

*Bacillus amyloliquefaciens*strain CD16 (KX348272) and *B*. *thuringiensis* strain EGU45 (DQ508971) were isolated in our laboratory [23]. These were grown on Himedia nutrient broth (NB) at 37°C with stirring at 200 rpm for 16 h. The media also contained CG (2%, v v^-1^) and the cultures were thus adapted to the substrate for 5 cycles. The cultures were then used as inoculum at the rate of 10 μg cellular protein mL^-1^[6].

### Hydrogen production

#### Immobilization of cells on lignocellulosic support material

Coconut coir (CC) was dried and packed in PVC tubes to prepare cartridges (3 x 2 cm) containing 3 g coir each, as reported earlier [6]. These cartridges were used as support material for bacterial immobilization. Aspirator bottles (1.2 L) with working volume of 1.0 L were used to perform the experiments. In order to allow the growth of biofilm on cartridges, casein enzyme hydrolyzate (CEH) was used as a biofilm forming media [21]. In bottles containing different amounts of cartridges (5–15%, vv^-1^), 100 mL of CEH was added. Each cartridge occupied ~10 mL of the 1.0 L working volume used. Thus, 5, 10 and 15 cartridges were used for 5%, 10% and 15% CC reactors. Free floating (FF) cultures were used as controls. Inoculation with CG acclimatized cultures was done at the rate of 10 μg cell protein mL^-1^. Anaerobic conditions were maintained by flushing the reactors with argon gas. The bottles were incubated at 37°C for 24 h without shaking, to allow biofilm formation. Domestic waste water diluted with tap water in the ratio of 3:1 was used as H_2_ producing media (DWW). Minimal salts (KH_2_PO_4_- 1.5 g/L, NaCl- 0.25 g/L, NH_4_Cl- 0.5 g/L, MgSO_4_(1.0 M)- 0.5 mL/L, CaCl_2_(1.0 M)- 0.5 mL/L) were added to the DWW media. After 24 h biofilm growth on cartridges in aspirator bottles, DWW containing CG (2%, v v^-1^) was used to complete the remaining working volume.

#### Batch culture

The aspirator bottles containing hydrogen producers immobilized on CC (5–15%, vv^-1^) in CG supplemented DWW media were made air tight using glass stoppers after adjusting the pH to 7.0. The pH was adjusted using NaOH (2.0 N) or HCl (2.0 N) after which argon flushing was given to maintain anaerobic environment inside the aspirator bottles. These were incubated at 37°C. A provision for gas outlet and liquid sampling was provided in the bottles. On a daily basis, the gas evolved was collected and analyzed. Adjustment of pH and argon flushing was also done on a daily basis until the gas production ceased. After this the fermentation was switched to continuous mode.

#### Continuous culture

For the continuous culture digestion, a hydraulic retention time (HRT) of 2 days was used. On a daily basis, 500 mL of the effluent was removed from each reactor and was replenished with fresh DWWTW media containing CG (2%, v v^-1^). Adjustment of pH and argon flushing was done daily, and the evolved gas was analyzed. Incubation was done at 37°C and the process was continued for 60 days to obtain steady gas production. The experiments were performed in triplicates.

#### Recycling of effluent

During the continuous H_2_ production effluent was generated daily. This effluent was further used for H_2_ production by mixing it with fresh DWW medium indifferent ratios: (i) 1:3, (ii) 1:1, and (iii) 3:1. The gas production was compared with the controls and the process was continued for an additional 60 days. The support material used in all these reactors contained 15%(v v^-1^) CC.

### Analytical methods

#### Gas analysis

Water displacement method was used to determine the volume of biogas produced. The composition of gas was analyzed using gas chromatography (Nucon GC5765, India) equipped with molecular sieve and Porapak-Q columns (1.8 m long and 2 mm inner diameter) and a thermal conductivity detector, as reported earlier [6, 24]. For the daily fed culture experiments, H_2_ yields were calculated on the glycerol fed basis.

#### Glycerol estimation

The amount of residual glycerol in the fermented medium was estimated by taking 1 mL of the sample. It was centrifuged at 10,000 g for 5 min. Supernatant (1μL) was injected into Gas chromatograph (Nucon GC5765, India) and analyzed under standard conditions as described earlier [6].

## Results and discussion

The effectiveness of biofilm forming *B*. *amyloliquefaciens* strain CD16 for a high and steady continuous H_2_ production has been reported [23]. However, the medium used in these studies is sterile distilled water. This increases the production cost for bio-energy generation. The medium thus used in present study is unsterile domestic waste diluted with tap water.

### Cell immobilization

Several support materials have been widely used for bacterial immobilization. These may include activated carbon, alginate gel, polyester fiber, porous glass beads, egg shells and lignocellulosic wastes such as banana leaves, groundnut shells, coconut coir, bamboo stem, etc.[25–28]. Apart from these, biofilms as natural cell entrapment strategy has also gained importance. Biofilms although have been extensively utilized for bioremediation is also gaining interest with bioenergy production [29, 30]. With the availability of medium that can screen biofilm formers, biofilm forming H_2_ producers have been isolated [21, 23]. One of these biofilm formers has been utilized for H_2_ production using wastewater in the present study. After a 24 h incubation, in the reactors inoculated with *B*. *amyloliquefaciens* strain CD16, biofilm formation was observed on CC cartridges. While in case of *B*. *thuringiensis* strain EGU45 no biofilm was formed. The cells immobilized in biofilm are resistant to environmental stresses and thus, may provide a better robust environment for gas production.

### Batch culture H_2_ production

The total biogas produced during 5 days batch fermentation by *B*. *thuringiensis* strain EGU45 ranged from 2.0 L to 3.1 L. The biogas constituted a mixture of H_2_ and CO_2_. The H_2_ in the produced gas constituted 56.2–70.2%. With *B*. *amyloliquefaciens* strain CD16, 2.4 L- 3.3 L biogas was produced which consisted of 58.3–60.0% H_2_ (**Table 1**). When comparing the H_2_ yield, 55 L H_2_/L CG to 110 L H_2_/L CG was produced by *B*. *thuringiensis* strain EGU45 while with *B*. *amyloliquefaciens* strain CD16, 70 L H_2_/L CG—100 L H_2_/L CG was obtained. The results obtained with DWWTW were strikingly similar to that obtained with sterile M-9 medium [23]. This shows that there are no deteriorating effects of using unsterile waste water as medium for biogas production under batch conditions.

**Table 1:** Hydrogen production from sewage water and crude glycerol by different *Bacillus* species: Batch culture.

Support material^a^ (%)	Biogas^b^ (mL)	Hydrogen		Yield^c^
		Volume(mL)	%	
*Bacillus thuringiensis* EGU45				
0	2000	1125	56.2	0.22
5	2850	1755	61.5	0.35
10	2945	1775	60.2	0.35
15	3140	2205	70.2	0.44
*Bacillus amyloliquefaciens* CD16				
0	2400	1400	58.3	0.28
5	3180	1930	60.0	0.38
10	3370	2015	59.7	0.40
15	3000	1925	64.1	0.38

^a^coconut coir.

^**b**^mixture of H_2_ + CO_2_.

^**c**^mol mol-1 crude glycerol utilized.

Feed: Sewage water medium diluted with Tap water in 3:1 ratio (M-9 salts: 0.5 X) with crude glycerol (2%, v/v). Inoculum: 10 μg cell protein mL^-1^ feed. Values represent 5 days of batch fermentation. All experiments were performed in triplicate. The standard deviation was less than 10%

### Continuous culture H_2_ production

For an economical large-scale bioenergy production, continuous culture fermentation is required. However, despite being an ideal system for higher product yields, cell washout is a major concern of this mode of fermentation. To deal with the problem, a number of bacterial support materials have been utilized. Recently, 1.18-fold increase in H_2_ production by using biofilm immobilized on lignocellulosic wastes has been reported [23].

**Fig 1:**
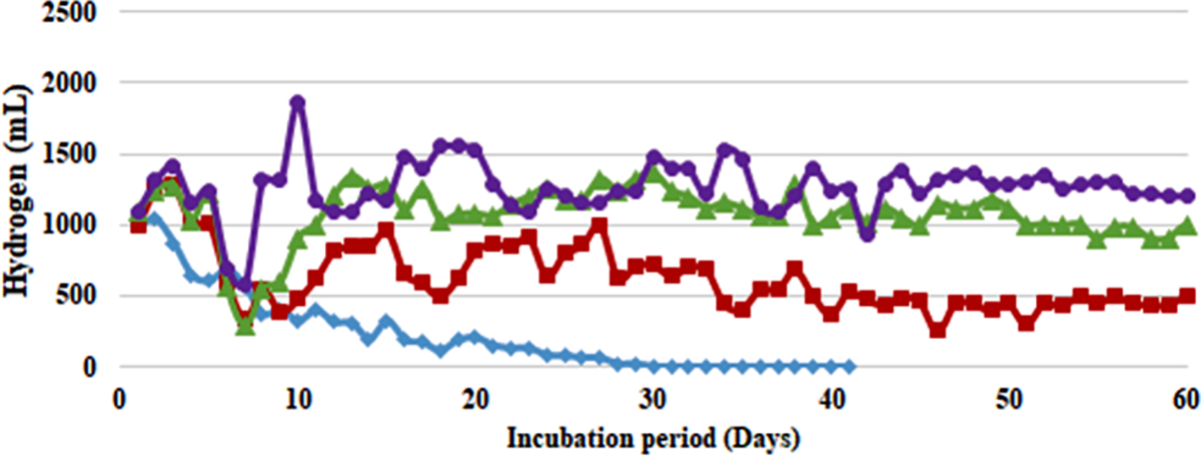
Continuous culture hydrogen production from sewage water and crude glycerol by *Bacillus thuringiensis* immobilized on coconut coir (CC): 5% (■, red filled square), 10% (▲, green filled triangle), 15% (●, violet filled circle) and Control (♦, blue filled diamond). Feed: 500 mL of Sewage water + Tap water in 3:1 ratio (0.05X M-9 salts) supplemented with crude glycerol (2%, v/v) at Hydraulic Retention Time of 2 days.

**Fig 2:**
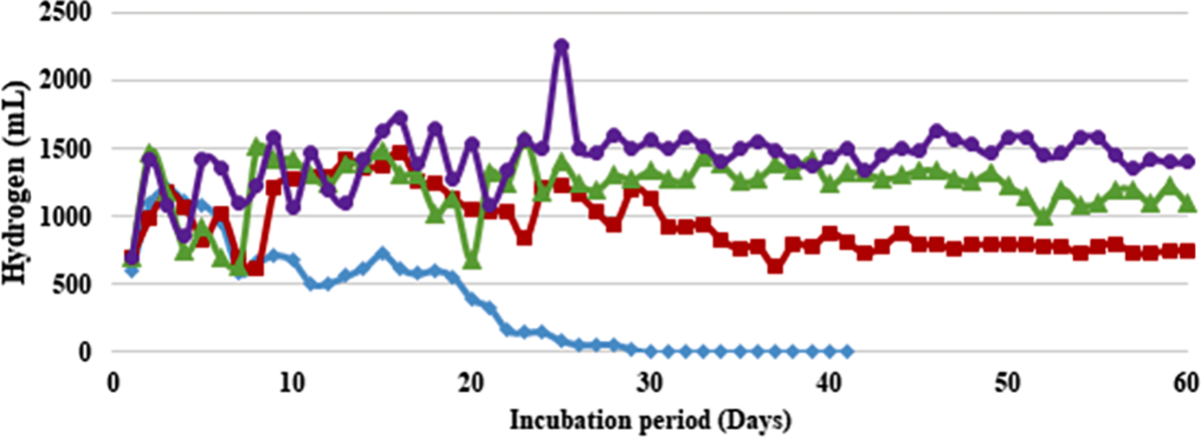
Continuous culture hydrogen production from sewage water and crude glycerol by *Bacillus amyloliquefaciens* immobilized on coconut coir (CC): 5% (■, red filled square), 10% (▲, green filled triangle), 15% (●, violet filled circle) and control (♦, blue filled diamond). Feed: 500 mL of Sewage water + Tap water in 3:1 ratio (0.05X M-9 salts) supplemented with crude glycerol (2%, v/v) at Hydraulic Retention Time of 2 days.

Similar strategy when applied to prevent cell washout from waste water medium in the current work resulted in encouragingly higher H_2_ yields during 60 days continuous fermentation. Without any cell immobilization, i.e. FF conditions, the biogas production showed a significant decline with both the strains (**Figs 1 and 2, and S1 and S2 Tables**). In case of *B*. *thuringiensis* strain EGU45, from an average of 0.6 L H_2_/ 0.5 L feed/day during initial 10 days of fermentation, the production declined to 0.06 L H_2_/ 0.5 L feed/day at the end of 30 days of fermentation. The gas production thereafter became so low that the reactors had to be terminated (**Table 2**). Similar was the case observed with biofilm forming *B*. *amyloliquefaciens* strain CD16, where the production declined from 0.8 L H_2_/ 0.5 L feed/day to 0.07 L H_2_/ 0.5 L feed/day during 30 days of fermentation and ceased thereafter (**Table 2**).

**Table 2:** Hydrogen production from sewage water and crude glycerol by different *Bacillus* species immobilized on lignocellulosic waste and the effect recycling of the effluent: Continuous culture.

	**Support material^a^ (%)**											
	**0**			**5**			**10**			**15**		
**DAI**	**Vol**^b^	**%**	**Yield**^c^	**Vol**^a^	**%**	**Yield**^b^	**Vol**^b^	**%**	**Yield**^c^	**Vol**^b^	**%**	**Yield**^c^
***Bacillus thuringiensis* EGU45**												
**0–10**	595	41.0	0.23	755	58.7	0.30	865	62.4	0.34	1200	65.2	0.48
**11–20**	215	37.0	0.08	755	58.3	0.30	1165	64.0	0.46	1330	64.4	0.53
**21–30**	60	57.1	0.02	780	56.1	0.31	1245	65.6	0.50	1225	59.4	0.49
**31–40**	-	-	-	545	54.5	0.21	1115	65.0	0.44	1305	66.5	0.52
**41–50**	-	-	-	415	44.6	0.16	1085	64.3	0.43	1265	59.1	0.50
**51–60**	-	-	-	465	56.3	0.18	955	64.0	0.38	1265	63.5	0.50
***Bacillus amyloliquefaciens* CD16**												
**0–10**	860	60.7	0.34	1015	59.7	0.40	1130	62.9	0.45	1255	62.5	0.50
**11–20**	550	67.4	0.22	1260	63.3	0.50	1230	62.5	0.49	1395	64.2	0.56
**21–30**	75	62.5	0.03	1065	59.4	0.42	1300	61.1	0.52	1580	65.8	0.63
**31–40**	-	-	-	810	57.8	0.32	1330	62.4	0.53	1475	63.8	0.59
**41–50**	-	-	-	790	64.2	0.31	1280	62.7	0.51	1510	63.7	0.60
**51–60**	-	-	-	760	72.0	0.30	1120	61.8	0.45	1450	66.2	0.58
	**Effluent recycling (%)**											
**DAI**	**0**			**25**			**50**			**75**		
***Bacillus thuringiensis* EGU45**												
**0–10**	1260	61.7	0.50	1345	65.7	0.54	1040	71.2	0.41	620	74.2	0.24
**11–20**	1310	62.5	0.52	1170	58.7	0.47	680	57.6	0.27	265	60.2	0.10
**21–30**	1280	61.6	0.51	1135	58.2	0.45	345	57.5	0.13	100	55.5	0.04
**31–40**	1180	61.6	0.47	955	48.9	0.38	300	58.0	0.12	90	45.0	0.03
**41–50**	995	60.3	0.40	910	58.5	0.36	-	-	-	-	-	-
**51–60**	985	63.3	0.39	840	60.0	0.33	-	-	-	-	-	-
***Bacillus amyloliquefaciens* CD16**												
**0–10**	1445	60.4	0.58	1460	57.4	0.58	1185	54.6	0.47	840	54.3	0.33
**11–20**	1480	62.1	0.59	1265	57.2	0.50	940	56.4	0.37	370	52.4	0.14
**21–30**	1285	61.0	0.51	1110	58.2	0.44	390	53.4	0.15	130	52.0	0.05
**31–40**	1395	61.0	0.56	1030	59.7	0.41	55	55.0	0.02	200	45.9	0.08
**41–50**	1230	61.8	0.49	980	59.0	0.39	-	-	-	-	-	**-**
**51–60**	1200	64.0	0.48	1015	63.0	0.40	-	-	-	-	-	**-**

^a^coconut coir.

^**b**^mixture of H_2_ + CO_2_ in mL.

^**c**^mol mol-1 crude glycerol utilized.

DAI: Days after incubation. Feed: Sewage water medium diluted with Tap water in 3:1 ratio (M-9 salts: 0.5 X) with crude glycerol (2%, v/v). Inoculum: 10 μg cell protein mL^-1^ feed. Values represent 5 days of batch fermentation. All experiments were performed in triplicate. The standard deviation was less than 10%

Effect of support material on biogas production could be clearly seen with its increasing quantity from 5–15% CC (vv^-1^). At 5% CC, non-biofilm former *B*. *thuringiensis* strain EGU45 produced 0.7 L H_2_/ 0.5 L feed/day for 30 days after which it reduced to 0.4 L H_2_/ 0.5 L feed/day and continued till 60 days maintaining a stable yield of 0.16–0.18 mol H_2_/ mol CG. On increasing the support material to 10% CC, 0.8 L H_2_/ 0.5 L feed/day was observed during initial 10 days of continuous culture. It increased thereafter and maintained a stable value of around 0.95–1.2 L H_2_/ 0.5 L feed/day during 60 days fermentation. The average H_2_ production with 10% CC was 2.28-fold higher than with 5% CC (**Table 3**). On increasing the support material to 15% CC, a higher and stable H_2_ of 1.2–1.3 L/ 0.5 L feed/day was produced during 60 days continuous fermentation. This corresponds to 0.48–0.53 mol H_2_/mol CG which was 1.23-fold higher than with 10%CC. Similar effect of increasing gas production with support material was also seen with biofilm former *B*. *amyloliquefaciens* strain CD16. At 5% CC, 1.0–1.2 L H_2_/ 0.5 L feed/day was produced during 0–30 days of fermentation which achieved a steady 0.7–0.8 L H_2_/ 0.5 L feed/day during 31–60 days of fermentation. At 10% CC, the gas production maintained a steady biogas production constituting 61.1–62.9% H_2_ throughout 60 days of fermentation. The amount of H_2_ varied from 1.1–1.3 L/0.5 L feed/day. This was 1.55-fold higher than with 5% CC. On increasing the support material to 15% CC, 1.2–1.5 L H_2_/ 0.5 L feed/day was produced during 60 days of fermentation, which was 1.23-fold higher than with 10% CC.

**Table 3:** Summarized hydrogen production from sewage water and crude glycerol by different *Bacillus* species and effect of recycling of the effluent: Continuous culture.

	Biogas^b^	Hydrogen			Biogas^b^	Hydrogen		
		Vol^c^	%	Yield^c^		Vol^c^	%	Yield^c^
Support material^a^ (%)	*Bacillus thuringiensis* EGU45				*Bacillus amyloliquefaciens* CD16			
0	295	135	45.5	0.01	485	315	64.9	0.02
5	935	470	50.2	0.18	1235	815	65.9	0.32
10	1670	1075	64.3	0.43	2100	1265	60.2	0.50
15	2165	1325	61.2	0.53	2395	1555	64.9	0.62
Effluent recycling (%)	*Bacillus thuringiensis* EGU45				*Bacillus amyloliquefaciens* CD16			
0	1685	1040	61.7	0.41	2045	1285	62.8	0.51
25	1550	920	59.3	0.36	1715	1045	60.9	0.42
50	395	220	55.6	0.08	480	260	54.1	0.10
75	125	65	52.0	0.02	165	85	51.5	0.03

^a^coconut coir.

^**b**^mixture of H_2_ + CO_2_ in mL.

^c^volume in mL.

^**d**^mol mol-1 crude glycerol utilized.

Feed: Sewage water medium diluted with Tap water in 3:1 ratio (M-9 salts: 0.5 X) with crude glycerol (2%, v/v). Inoculum: 10μg cell protein mL^-1^ feed. Values are based on observations made over a period of last 20 days of fermentation in three replicates at an HRT of 2 days. The standard deviation was less than 10%

On comparing the H_2_ producing abilities of two strains, without any support material both the strains produced an abysmal low H_2_ (**Figs 1 and 2**). Immobilizing *B*. *thuringiensis* strain EGU45 on CC increased the H_2_ production by 4- to 10- fold. With biofilm forming *B*. *amyloliquefaciens* strain CD16, the increase in H_2_ production with CC was 3- to 5- fold. Biofilm forming strain at 15% CC resulted in 1.17 times more H_2_ as compared to non-biofilm forming strain (**Table 3**).

### Effect of effluent recycling

**Fig 3:**
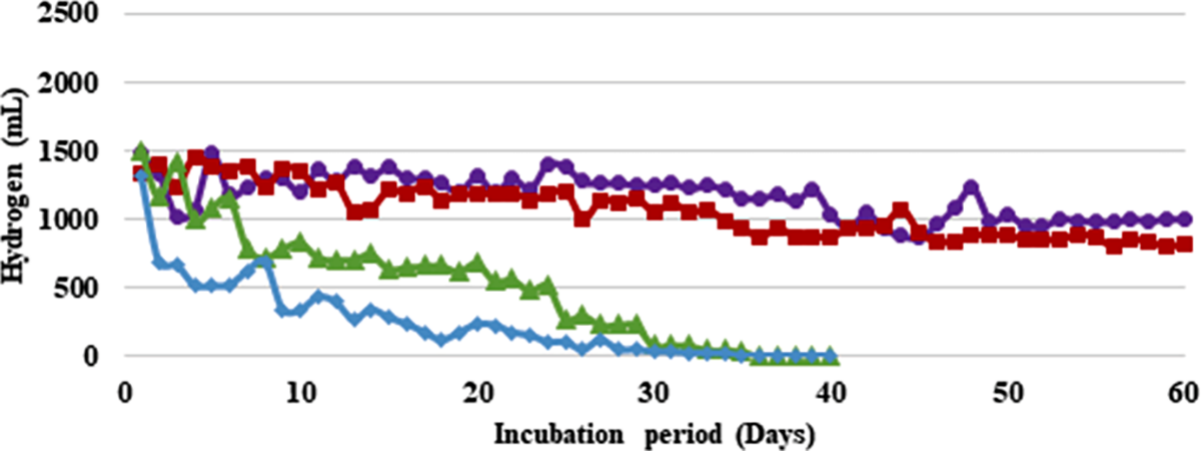
Effect of recycling of effluent on hydrogen production from sewage water and crude glycerol by *Bacillus thuringiensis* immobilized on coconut coir (CC): Recycling of effluent—25% (■, red filled square), 50% (▲, green filled triangle), 75% (♦, blue filled diamond) and control (●, violet filled circle). Feed: 500 mL of Sewage water + Tap water in 3:1 ratio (0.05X M-9 salts) supplemented with crude glycerol (2%, v/v) in case of control and 125–375 mL Sewage water + Tap water in 3:1 ratio (0.05X M-9 salts) made up to 500 mL with effluent in other cases.

**Fig 4:**
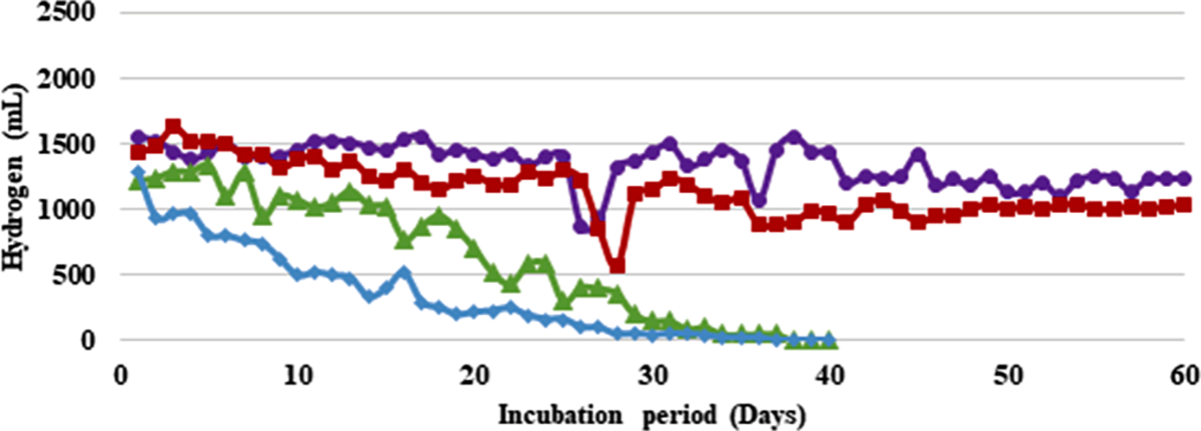
Effect of recycling of effluent on hydrogen production from crude glycerol by *Bacillus amyloliquefaciens* immobilized on coconut coir (CC): Recycling of effluent—25% (■, red filled square), 50% (▲, green filled triangle), 75% (♦, blue filled diamond) and control (●, violet filled circle). Feed: 500 mL of Sewage water + Tap water in 3:1 ratio (0.05X M-9 salts) supplemented with crude glycerol (2%, v/v) in case of control and 125–375 mL Sewage water + Tap water in 3:1 ratio (0.05X M-9 salts) made up to 500 mL with effluent in other cases.

To increase the overall process efficiency and economy, effluent generated from the H_2_ production stage was recycled. With, *B*. *thuringiensis* strain EGU45, at 75% and 50% effluent recycling a very sharp decline in gas production was recorded (**Figs 3 and 4 and S3 and S4 Tables**). After an initial production of 0.6–1.0 L H_2_/ 0.5 L feed/day in these cases, the H_2_ production declined to 0.09–0.3 L H_2_/ 0.5 L feed/day and ceased thereafter. However, at 25% effluent recycling a significant difference was not observed with respect to control during 60 days of fermentation. During initial 20 days of recycling, 1.2–1.4 L H_2_/ 0.5 L feed/day was observed. After this a small decline in gas production was observed which maintained an average of 0.9 LH_2_/ 0.5 L feed/day till 60 days of recycling (**Table 2**). Considering the average gas produced, a 6% drop in H_2_ was observed with 25% effluent recycling as compared to controls This corresponded to 0.36 mol H_2_/mol CG as compared to controls which produced 0.41 mol H_2_/mol CG (**Table 3**). With *B*. *amyloliquefaciens* strain CD16, a similar trend of sharp decline in gas production was observed at 75% and 50% recycling. The gas production declined to 0.2 L H_2_/ 0.5 L feed/day and 0.05 L H_2_/ 0.5 L feed/day at 75% and 50% recycling respectively within 40 days of recycling and ceased thereafter. However, at 25% recycling the gas production did not show any significant reduction. An average of 1.0 L H_2_/ 0.5 L feed/day was produced with 25% recycling which was a 10% decline as compared to controls (**Table 3**).The reactors with 15% CC support material were run for 120 days (an additional 60 days during recycling of effluent) for the entire duration of which an average H_2_ yield of 100–120 L/L CG was maintained by both the strains (**Tables 2 and 3**).

## Conclusion

Waste generation is an integral part of our routine activities. Under natural environmental conditions, microbes metabolize the organic matter content and release gases into the atmosphere. This contributes significantly to environmental pollution. Another contributor to environmental pollution is the burning of fossil fuels. Efforts to treat biowastes through microbial activity have revealed that this concept can be exploited to produce energy rich gases (H_2_ and methane, CH_4_) through fermentation. A wide range of biowastes have the potential to produce H_2_ and CH_4_. A major limitation in the use of H_2_ producers is the risk of contamination which emanates from bacteria present in the unsterile biowastes. So to avoid contamination, sterilization become imperative, this obviously results in lowering economic efficiency. Secondly, in most biological processes, the substrate concentrations vary from very low of 0.1% to a maximum 10%. It implies that 90 to 99.9% is water or medium. In this study, we have circumvented almost all the issues related to biological H_2_ production: (i) use of unsterile conditions, (ii) use of sewage water as medium, (iii) use crude glycerol, which otherwise cause heavy pollution, (iii) use of a single bacterium with abilities to form biofilm and produce H_2_, (iv) recycling of the effluent for further enhancing the process efficiency, (v) continuous culture conditions enable easy operation (vi) no stirring required, (vii) independent of light, and (viii) used organism i.e. *Bacillus*, which has been categorized as GRAS (Generally Regarded As Safe) organism [31]. Biofilm forming bacteria *B*. *amyloliquefaciens* CD16 immobilized on CC utilized CG in domestic wastewater medium to produce 120 L H_2_/L CG during 120 days continuous fermentation. Under similar conditions, the non-biofilm forming *B*. *thuringiensis* strain EGU45 produced around 100 L H_2_/L CG fed (This study). In contrast, there are only a limited number of studies where wastewater has been co-digested with CG (1% w/v) by *Klebsiella* sp. It generated around 9.8 L H_2_/g substrate consumed, with H_2_ constituting only 44% of the total biogas [32]. Using activated sludge from biodiesel industry effluent, 75L H_2_/L glycerol consumed was reported in anaerobic sequencing batch reactors, with H_2_ constituting only 33.4% of the total biogas produced [22]. Further, we have observed that on recycling the effluent up to 25%, no drastic changes in gas production were observed. In comparison to the use of sterile distilled water as medium, the use of wastewater did not result in any adverse effect on H_2_ production. A similar response on recycling of effluent from H2 production stage was shown in our previous study, where sterile distilled water was used for preparing the slurry [23]. Another interesting aspect of this study is the possibility of further utilization of effluent of H_2_ production stage for producing value added products such as polyhydroxyalkanoates and CH_4_ [14, 18].

## Supporting information

S1 Table Effect of support material on continuous culture hydrogen production by *Bacillus thuringiensis* EGU45. (DOCX)

S2 Table Effect of support material on continuous culture hydrogen production by *Bacillus amyloliquefaciens* CD16. (DOCX)

S3 Table Effect of Effluent recycling on continuous culture hydrogen production by *Bacillus thuringiensis* EGU45. (DOCX)

S4 Table Effect of Effluent recycling on continuous culture hydrogen production by *Bacillus amyloliquefaciens* CD16. (DOCX)
