# Supplementary material for: Bio-hydrogen production by co-digestion of domestic wastewater and biodiesel industry effluent
Source: PLoS One. 2018 Jul 11;13(7):e0199059. doi: 10.1371/journal.pone.0199059 (PMC6040696; doi:10.1371/journal.pone.0199059)
Supplement: S1 Table — (DOCX) [file pone.0199059.s001.docx]

**S1 Table: Effect of support material on continuous culture hydrogen production by *Bacillus thuringiensis* EGU45**

| **DAI** | **Biogas (mL)** | **Hydrogen** | | **Yield** |
| --- | --- | --- | --- | --- |
|  |  | **Volume (mL)** | **%** |  |
| **Free Floating** | | | | |
| 1 | 2095 | 1045 | 49.8 | 0.42 |
| 2 | 2190 | 875 | 39.9 | 0.35 |
| 3 | 1715 | 650 | 37.9 | 0.26 |
| 4 | 1620 | 610 | 37.6 | 0.24 |
| 5 | 1715 | 690 | 40.2 | 0.27 |
| 6 | 1430 | 570 | 39.8 | 0.22 |
| 7 | 950 | 375 | 39.4 | 0.15 |
| 8 | 950 | 405 | 42.6 | 0.16 |
| 9 | 855 | 325 | 38.0 | 0.13 |
| 10 | 950 | 400 | 42.1 | 0.16 |
| 11 | 855 | 315 | 36.8 | 0.12 |
| 12 | 760 | 310 | 40.7 | 0.12 |
| 13 | 475 | 195 | 41.0 | 0.07 |
| 14 | 855 | 325 | 38.0 | 0.13 |
| 15 | 475 | 190 | 40.0 | 0.07 |
| 16 | 475 | 170 | 35.7 | 0.06 |
| 17 | 570 | 115 | 20.1 | 0.04 |
| 18 | 475 | 190 | 40.0 | 0.07 |
| 19 | 475 | 205 | 43.1 | 0.08 |
| 20 | 380 | 150 | 39.4 | 0.06 |
| 21 | 235 | 130 | 55.3 | 0.05 |
| 22 | 235 | 130 | 55.3 | 0.05 |
| 23 | 140 | 80 | 57.1 | 0.03 |
| 24 | 140 | 75 | 53.5 | 0.03 |
| 25 | 95 | 65 | 68.4 | 0.02 |
| 26 | 95 | 60 | 63.1 | 0.02 |
| 27 | 45 | 20 | 44.4 | 0.008 |
| 28 | 45 | 20 | 44.4 | 0.008 |
| 29 | 0 | 0 | N.A | N.A |
| 30 | 0 | 0 | N.A | N.A |
| 31 | 0 | 0 | N.A | N.A |
| 32 | 0 | 0 | N.A | N.A |
| 33 | 0 | 0 | N.A | N.A |
| 34 | 0 | 0 | N.A | N.A |
| 35 | 0 | 0 | N.A | N.A |
| 36 | 0 | 0 | N.A | N.A |
| 37 | 0 | 0 | N.A | N.A |
| 38 | 0 | 0 | N.A | N.A |
| 39 | 0 | 0 | N.A | N.A |
| 40 | 0 | 0 | N.A | N.A |
| 41 | 0 | 0 | N.A | N.A |
| 42 | 0 | 0 | N.A | N.A |
| 43 | 0 | 0 | N.A | N.A |
| 44 | 0 | 0 | N.A | N.A |
| 45 | 0 | 0 | N.A | N.A |
| 46 | 0 | 0 | N.A | N.A |
| 47 | 0 | 0 | N.A | N.A |
| 48 | 0 | 0 | N.A | N.A |
| 49 | 0 | 0 | N.A | N.A |
| 50 | 0 | 0 | N.A | N.A |
| 51 | 0 | 0 | N.A | N.A |
| 52 | 0 | 0 | N.A | N.A |
| 53 | 0 | 0 | N.A | N.A |
| 54 | 0 | 0 | N.A | N.A |
| 55 | 0 | 0 | N.A | N.A |
| 56 | 0 | 0 | N.A | N.A |
| 57 | 0 | 0 | N.A | N.A |
| 58 | 0 | 0 | N.A | N.A |
| 59 | 0 | 0 | N.A | N.A |
| 60 | 0 | 0 | N.A | N.A |
| **Coconut coir (5%)** | | | | |
| 1 | 2095 | 1270 | 60.6 | 0.51 |
| 2 | 2185 | 1280 | 58.5 | 0.51 |
| 3 | 1715 | 1010 | 58.8 | 0.40 |
| 4 | 1715 | 1010 | 58.8 | 0.40 |
| 5 | 855 | 595 | 69.5 | 0.23 |
| 6 | 520 | 340 | 65.3 | 0.13 |
| 7 | 950 | 550 | 57.8 | 0.22 |
| 8 | 715 | 390 | 54.5 | 0.15 |
| 9 | 950 | 485 | 51.0 | 0.19 |
| 10 | 1140 | 625 | 54.8 | 0.25 |
| 11 | 1430 | 820 | 57.3 | 0.32 |
| 12 | 1430 | 845 | 59.0 | 0.33 |
| 13 | 1525 | 845 | 55.4 | 0.33 |
| 14 | 1620 | 960 | 59.2 | 0.38 |
| 15 | 1140 | 660 | 57.8 | 0.26 |
| 16 | 950 | 590 | 62.1 | 0.23 |
| 17 | 855 | 500 | 58.4 | 0.20 |
| 18 | 1140 | 630 | 55.2 | 0.25 |
| 19 | 1430 | 820 | 57.3 | 0.32 |
| 20 | 1430 | 870 | 60.8 | 0.34 |
| 21 | 1520 | 850 | 55.9 | 0.34 |
| 22 | 1620 | 910 | 56.1 | 0.36 |
| 23 | 1140 | 645 | 56.5 | 0.25 |
| 24 | 1430 | 800 | 55.9 | 0.32 |
| 25 | 1525 | 860 | 56.3 | 0.34 |
| 26 | 1715 | 1000 | 58.3 | 0.40 |
| 27 | 1140 | 630 | 55.2 | 0.25 |
| 28 | 1240 | 715 | 57.6 | 0.28 |
| 29 | 1240 | 720 | 58.0 | 0.28 |
| 30 | 1330 | 645 | 48.4 | 0.25 |
| 31 | 1140 | 705 | 61.8 | 0.28 |
| 32 | 1140 | 685 | 60.0 | 0.27 |
| 33 | 760 | 445 | 58.5 | 0.17 |
| 34 | 665 | 410 | 61.6 | 0.16 |
| 35 | 950 | 550 | 57.8 | 0.22 |
| 36 | 950 | 550 | 57.8 | 0.22 |
| 37 | 1140 | 685 | 60.0 | 0.27 |
| 38 | 1140 | 505 | 44.2 | 0.20 |
| 39 | 950 | 370 | 38.9 | 0.14 |
| 40 | 1140 | 530 | 46.4 | 0.21 |
| 41 | 1140 | 475 | 41.6 | 0.19 |
| 42 | 1140 | 440 | 38.5 | 0.17 |
| 43 | 1240 | 490 | 39.5 | 0.19 |
| 44 | 1140 | 465 | 40.7 | 0.18 |
| 45 | 760 | 250 | 32.8 | 0.10 |
| 46 | 855 | 450 | 52.6 | 0.18 |
| 47 | 665 | 450 | 67.6 | 0.18 |
| 48 | 760 | 400 | 52.6 | 0.16 |
| 49 | 855 | 450 | 52.6 | 0.18 |
| 50 | 760 | 300 | 39.4 | 0.12 |
| 51 | 760 | 450 | 59.2 | 0.18 |
| 52 | 855 | 430 | 50.2 | 0.17 |
| 53 | 855 | 500 | 58.4 | 0.20 |
| 54 | 760 | 450 | 59.2 | 0.18 |
| 55 | 760 | 500 | 65.7 | 0.20 |
| 56 | 855 | 450 | 52.6 | 0.18 |
| 57 | 855 | 440 | 51.4 | 0.17 |
| 58 | 855 | 440 | 51.4 | 0.17 |
| 59 | 855 | 500 | 58.4 | 0.20 |
| 60 | 855 | 500 | 58.4 | 0.20 |
| **Coconut coir (10%)** | | | | |
| 1 | 2000 | 1240 | 62.0 | 0.49 |
| 2 | 1905 | 1275 | 66.9 | 0.51 |
| 3 | 1810 | 1030 | 56.9 | 0.41 |
| 4 | 2000 | 1220 | 61.0 | 0.49 |
| 5 | 905 | 555 | 61.3 | 0.22 |
| 6 | 570 | 285 | 50.0 | 0.11 |
| 7 | 855 | 550 | 64.3 | 0.22 |
| 8 | 855 | 600 | 70.1 | 0.24 |
| 9 | 1430 | 905 | 63.2 | 0.36 |
| 10 | 1525 | 1000 | 65.5 | 0.40 |
| 11 | 1810 | 1200 | 66.2 | 0.48 |
| 12 | 2095 | 1340 | 63.9 | 0.53 |
| 13 | 1905 | 1255 | 65.8 | 0.50 |
| 14 | 1905 | 1275 | 66.9 | 0.51 |
| 15 | 1810 | 1105 | 61.0 | 0.44 |
| 16 | 2095 | 1255 | 59.9 | 0.50 |
| 17 | 1715 | 1030 | 60.0 | 0.41 |
| 18 | 1620 | 1070 | 66.0 | 0.43 |
| 19 | 1620 | 1075 | 66.3 | 0.43 |
| 20 | 1620 | 1060 | 65.4 | 0.42 |
| 21 | 1715 | 1140 | 64.1 | 0.45 |
| 22 | 1810 | 1195 | 66.4 | 0.48 |
| 23 | 1905 | 1260 | 66.0 | 0.50 |
| 24 | 1905 | 1180 | 66.1 | 0.47 |
| 25 | 1810 | 1175 | 61.9 | 0.47 |
| 26 | 2000 | 1320 | 64.9 | 0.53 |
| 27 | 1905 | 1240 | 66.0 | 0.49 |
| 28 | 2000 | 1320 | 65.0 | 0.53 |
| 29 | 2000 | 1360 | 66.0 | 0.54 |
| 30 | 1905 | 1240 | 68.0 | 0.49 |
| 31 | 1810 | 1195 | 66.0 | 0.48 |
| 32 | 1715 | 1115 | 65.0 | 0.44 |
| 33 | 1715 | 1150 | 67.0 | 0.46 |
| 34 | 1715 | 1115 | 65.0 | 0.44 |
| 35 | 1620 | 1055 | 65.1 | 0.42 |
| 36 | 1620 | 1055 | 65.1 | 0.42 |
| 37 | 2000 | 1280 | 64.0 | 0.51 |
| 38 | 1620 | 1000 | 61.7 | 0.40 |
| 39 | 1620 | 1050 | 64.8 | 0.42 |
| 40 | 1715 | 1115 | 65.0 | 0.44 |
| 41 | 1620 | 1020 | 62.9 | 0.40 |
| 42 | 1715 | 1115 | 65.0 | 0.44 |
| 43 | 1620 | 1050 | 64.8 | 0.42 |
| 44 | 1525 | 990 | 64.9 | 0.39 |
| 45 | 1810 | 1140 | 62.9 | 0.45 |
| 46 | 1715 | 1115 | 65.0 | 0.44 |
| 47 | 1715 | 1115 | 65.0 | 0.44 |
| 48 | 1810 | 1175 | 64.9 | 0.47 |
| 49 | 1715 | 1115 | 65.0 | 0.44 |
| 50 | 1620 | 1000 | 61.7 | 0.40 |
| 51 | 1525 | 1000 | 65.5 | 0.40 |
| 52 | 1525 | 990 | 64.9 | 0.39 |
| 53 | 1620 | 1000 | 61.7 | 0.40 |
| 54 | 1525 | 900 | 59.0 | 0.36 |
| 55 | 1430 | 980 | 68.5 | 0.39 |
| 56 | 1430 | 980 | 68.5 | 0.39 |
| 57 | 1525 | 900 | 59.0 | 0.36 |
| 58 | 1430 | 900 | 62.9 | 0.36 |
| 59 | 1430 | 990 | 69.2 | 0.39 |
| 60 | 1430 | 900 | 62.9 | 0.36 |
| **Coconut coir (15%)** | | | | |
| 1 | 2190 | 1100 | 50.2 | 0.44 |
| 2 | 2285 | 1315 | 57.5 | 0.52 |
| 3 | 1905 | 1415 | 74.2 | 0.56 |
| 4 | 1905 | 1160 | 60.8 | 0.46 |
| 5 | 1050 | 640 | 61.5 | 0.25 |
| 6 | 855 | 690 | 80.7 | 0.27 |
| 7 | 2000 | 980 | 49.0 | 0.39 |
| 8 | 2095 | 1310 | 62.5 | 0.52 |
| 9 | 2190 | 1310 | 59.8 | 0.52 |
| 10 | 1905 | 1160 | 60.8 | 0.46 |
| 11 | 1715 | 1180 | 68.8 | 0.47 |
| 12 | 1715 | 1100 | 64.1 | 0.44 |
| 13 | 1905 | 1100 | 57.7 | 0.44 |
| 14 | 1810 | 1220 | 67.4 | 0.49 |
| 15 | 2190 | 1175 | 53.6 | 0.47 |
| 16 | 2285 | 1480 | 64.7 | 0.59 |
| 17 | 2380 | 1390 | 58.4 | 0.55 |
| 18 | 2380 | 1550 | 65.1 | 0.62 |
| 19 | 2285 | 1550 | 67.8 | 0.62 |
| 20 | 2000 | 1530 | 76.5 | 0.61 |
| 21 | 2000 | 1290 | 64.5 | 0.51 |
| 22 | 1905 | 1140 | 59.8 | 0.45 |
| 23 | 2190 | 1085 | 49.5 | 0.43 |
| 24 | 2095 | 1260 | 60.1 | 0.50 |
| 25 | 2000 | 1205 | 60.2 | 0.48 |
| 26 | 1810 | 1150 | 63.5 | 0.46 |
| 27 | 1905 | 1160 | 60.8 | 0.46 |
| 28 | 2000 | 1240 | 62.0 | 0.49 |
| 29 | 2380 | 1240 | 52.1 | 0.49 |
| 30 | 2285 | 1470 | 64.3 | 0.59 |
| 31 | 2190 | 1405 | 64.1 | 0.56 |
| 32 | 2105 | 1390 | 66.0 | 0.55 |
| 33 | 2475 | 1220 | 49.2 | 0.49 |
| 34 | 2285 | 1530 | 66.9 | 0.61 |
| 35 | 1810 | 1460 | 80.6 | 0.58 |
| 36 | 1715 | 1120 | 65.3 | 0.45 |
| 37 | 1905 | 1085 | 56.9 | 0.43 |
| 38 | 2095 | 1200 | 57.2 | 0.48 |
| 39 | 2000 | 1395 | 69.7 | 0.56 |
| 40 | 2000 | 1240 | 62.0 | 0.49 |
| 41 | 1905 | 1255 | 65.8 | 0.50 |
| 42 | 2000 | 925 | 46.2 | 0.37 |
| 43 | 2905 | 1280 | 44.0 | 0.51 |
| 44 | 2000 | 1375 | 68.7 | 0.55 |
| 45 | 2095 | 1225 | 58.4 | 0.49 |
| 46 | 2095 | 1315 | 62.7 | 0.52 |
| 47 | 2190 | 1350 | 61.6 | 0.54 |
| 48 | 2000 | 1360 | 68.0 | 0.54 |
| 49 | 2095 | 1280 | 61.0 | 0.51 |
| 50 | 2095 | 1290 | 61.5 | 0.51 |
| 51 | 2190 | 1300 | 59.3 | 0.52 |
| 52 | 2000 | 1350 | 67.5 | 0.54 |
| 53 | 2000 | 1260 | 63.0 | 0.50 |
| 54 | 1905 | 1280 | 67.1 | 0.51 |
| 55 | 1905 | 1300 | 68.2 | 0.52 |
| 56 | 1905 | 1300 | 68.2 | 0.52 |
| 57 | 2000 | 1220 | 61.0 | 0.49 |
| 58 | 1905 | 1220 | 64.0 | 0.49 |
| 59 | 2095 | 1200 | 57.2 | 0.48 |
| 60 | 2000 | 1200 | 60.0 | 0.48 |
