# Supplementary material for: Bio-hydrogen production by co-digestion of domestic wastewater and biodiesel industry effluent
Source: PLoS One. 2018 Jul 11;13(7):e0199059. doi: 10.1371/journal.pone.0199059 (PMC6040696; doi:10.1371/journal.pone.0199059)
Supplement: S2 Table — (DOCX) [file pone.0199059.s002.docx]

**S2 Table: Effect of support material on continuous culture hydrogen production by *Bacillus amyloliquefaciens* CD16**

| **DAI** | **Biogas (mL)** | **Hydrogen** | | **Yield** |
| --- | --- | --- | --- | --- |
|  |  | **Volume (mL)** | **%** |  |
| **Free Floating** | | | | |
| 1 | 1715 | 1095 | 63.8 | 0.44 |
| 2 | 1905 | 1210 | 63.5 | 0.48 |
| 3 | 2000 | 1110 | 55.5 | 0.44 |
| 4 | 1905 | 1085 | 56.9 | 0.43 |
| 5 | 1520 | 955 | 62.8 | 0.38 |
| 6 | 1045 | 590 | 56.4 | 0.23 |
| 7 | 1045 | 660 | 63.1 | 0.26 |
| 8 | 1140 | 715 | 62.7 | 0.28 |
| 9 | 1045 | 675 | 64.5 | 0.27 |
| 10 | 855 | 510 | 59.6 | 0.20 |
| 11 | 855 | 510 | 59.6 | 0.20 |
| 12 | 950 | 565 | 59.4 | 0.22 |
| 13 | 950 | 625 | 65.7 | 0.25 |
| 14 | 1045 | 725 | 69.3 | 0.29 |
| 15 | 855 | 615 | 71.9 | 0.24 |
| 16 | 855 | 580 | 67.8 | 0.23 |
| 17 | 855 | 605 | 70.7 | 0.24 |
| 18 | 760 | 555 | 73.0 | 0.22 |
| 19 | 570 | 395 | 69.2 | 0.15 |
| 20 | 475 | 335 | 70.5 | 0.13 |
| 21 | 285 | 165 | 57.8 | 0.06 |
| 22 | 235 | 150 | 63.8 | 0.06 |
| 23 | 235 | 150 | 63.8 | 0.06 |
| 24 | 140 | 90 | 64.2 | 0.03 |
| 25 | 95 | 60 | 63.1 | 0.02 |
| 26 | 95 | 60 | 63.1 | 0.02 |
| 27 | 95 | 60 | 63.1 | 0.02 |
| 28 | 45 | 25 | 55.5 | 0.01 |
| 29 | 0 | 0 | N.A | N.A |
| 30 | 0 | 0 | N.A | N.A |
| 31 | 0 | 0 | N.A | N.A |
| 32 | 0 | 0 | N.A | N.A |
| 33 | 0 | 0 | N.A | N.A |
| 34 | 0 | 0 | N.A | N.A |
| 35 | 0 | 0 | N.A | N.A |
| 36 | 0 | 0 | N.A | N.A |
| 37 | 0 | 0 | N.A | N.A |
| 38 | 0 | 0 | N.A | N.A |
| 39 | 0 | 0 | N.A | N.A |
| 40 | 0 | 0 | N.A | N.A |
| 41 | 0 | 0 | N.A | N.A |
| 42 | 0 | 0 | N.A | N.A |
| 43 | 0 | 0 | N.A | N.A |
| 44 | 0 | 0 | N.A | N.A |
| 45 | 0 | 0 | N.A | N.A |
| 46 | 0 | 0 | N.A | N.A |
| 47 | 0 | 0 | N.A | N.A |
| 48 | 0 | 0 | N.A | N.A |
| 49 | 0 | 0 | N.A | N.A |
| 50 | 0 | 0 | N.A | N.A |
| 51 | 0 | 0 | N.A | N.A |
| 52 | 0 | 0 | N.A | N.A |
| 53 | 0 | 0 | N.A | N.A |
| 54 | 0 | 0 | N.A | N.A |
| 55 | 0 | 0 | N.A | N.A |
| 56 | 0 | 0 | N.A | N.A |
| 57 | 0 | 0 | N.A | N.A |
| 58 | 0 | 0 | N.A | N.A |
| 59 | 0 | 0 | N.A | N.A |
| 60 | 0 | 0 | N.A | N.A |
| **Coconut coir (5%)** | | | | |
| 1 | 2000 | 985 | 49.2 | 0.39 |
| 2 | 2095 | 1175 | 56.0 | 0.47 |
| 3 | 1715 | 1060 | 61.8 | 0.42 |
| 4 | 1430 | 830 | 58.0 | 0.33 |
| 5 | 1715 | 1020 | 59.4 | 0.40 |
| 6 | 1095 | 690 | 63.0 | 0.27 |
| 7 | 1045 | 620 | 59.3 | 0.24 |
| 8 | 1905 | 1215 | 63.7 | 0.48 |
| 9 | 1905 | 1270 | 66.6 | 0.51 |
| 10 | 2095 | 1295 | 61.8 | 0.52 |
| 11 | 2095 | 1295 | 61.8 | 0.52 |
| 12 | 2285 | 1415 | 61.9 | 0.56 |
| 13 | 2095 | 1360 | 64.9 | 0.54 |
| 14 | 2190 | 1375 | 62.7 | 0.55 |
| 15 | 2285 | 1475 | 64.5 | 0.59 |
| 16 | 2000 | 1260 | 63.0 | 0.50 |
| 17 | 1905 | 1235 | 64.8 | 0.49 |
| 18 | 1715 | 1130 | 65.8 | 0.45 |
| 19 | 1620 | 1045 | 64.5 | 0.42 |
| 20 | 1715 | 1030 | 60.0 | 0.41 |
| 21 | 1905 | 1030 | 54.0 | 0.41 |
| 22 | 1525 | 835 | 54.7 | 0.33 |
| 23 | 2095 | 1210 | 57.7 | 0.48 |
| 24 | 2000 | 1220 | 61.0 | 0.49 |
| 25 | 2000 | 1155 | 57.7 | 0.46 |
| 26 | 1715 | 1030 | 60.0 | 0.41 |
| 27 | 1620 | 940 | 58.0 | 0.37 |
| 28 | 1715 | 1200 | 69.9 | 0.48 |
| 29 | 1715 | 1125 | 65.5 | 0.45 |
| 30 | 1620 | 925 | 57.0 | 0.37 |
| 31 | 1525 | 925 | 60.6 | 0.37 |
| 32 | 1620 | 940 | 58.0 | 0.37 |
| 33 | 1430 | 820 | 57.3 | 0.32 |
| 34 | 1330 | 765 | 57.5 | 0.30 |
| 35 | 1240 | 780 | 62.9 | 0.31 |
| 36 | 1140 | 630 | 55.2 | 0.25 |
| 37 | 1430 | 800 | 55.9 | 0.32 |
| 38 | 1330 | 775 | 58.2 | 0.31 |
| 39 | 1520 | 880 | 57.8 | 0.35 |
| 40 | 1430 | 810 | 56.6 | 0.32 |
| 41 | 1240 | 730 | 58.8 | 0.29 |
| 42 | 1240 | 780 | 62.9 | 0.31 |
| 43 | 1430 | 875 | 61.1 | 0.35 |
| 44 | 1240 | 795 | 64.1 | 0.31 |
| 45 | 1240 | 795 | 64.1 | 0.31 |
| 46 | 1240 | 755 | 60.8 | 0.30 |
| 47 | 1330 | 790 | 59.3 | 0.31 |
| 48 | 1140 | 790 | 69.2 | 0.31 |
| 49 | 1140 | 790 | 69.2 | 0.31 |
| 50 | 1045 | 790 | 75.5 | 0.31 |
| 51 | 1045 | 780 | 74.6 | 0.31 |
| 52 | 1140 | 780 | 68.4 | 0.31 |
| 53 | 1140 | 730 | 64.0 | 0.29 |
| 54 | 1045 | 780 | 74.6 | 0.31 |
| 55 | 950 | 800 | 84.2 | 0.32 |
| 56 | 950 | 730 | 76.8 | 0.29 |
| 57 | 950 | 730 | 76.8 | 0.29 |
| 58 | 1045 | 750 | 71.7 | 0.30 |
| 59 | 1140 | 750 | 65.7 | 0.30 |
| 60 | 1140 | 750 | 65.7 | 0.30 |
| **Coconut coir (10%)** | | | | |
| 1 | 2380 | 1475 | 61.9 | 0.59 |
| 2 | 2095 | 1175 | 56.0 | 0.47 |
| 3 | 1240 | 745 | 60.0 | 0.29 |
| 4 | 1430 | 915 | 63.9 | 0.36 |
| 5 | 1095 | 690 | 63.0 | 0.27 |
| 6 | 1045 | 640 | 61.2 | 0.25 |
| 7 | 2285 | 1510 | 66.0 | 0.60 |
| 8 | 2190 | 1425 | 65.0 | 0.57 |
| 9 | 2190 | 1425 | 65.0 | 0.57 |
| 10 | 2000 | 1300 | 65.0 | 0.52 |
| 11 | 1905 | 1240 | 65.0 | 0.49 |
| 12 | 2095 | 1395 | 66.5 | 0.56 |
| 13 | 2190 | 1390 | 63.4 | 0.55 |
| 14 | 2285 | 1485 | 64.9 | 0.59 |
| 15 | 2190 | 1315 | 60.0 | 0.52 |
| 16 | 2095 | 1300 | 62.0 | 0.52 |
| 17 | 1620 | 1025 | 63.2 | 0.41 |
| 18 | 2095 | 1130 | 53.9 | 0.45 |
| 19 | 1095 | 685 | 62.5 | 0.27 |
| 20 | 2095 | 1325 | 63.2 | 0.53 |
| 21 | 2000 | 1250 | 62.5 | 0.50 |
| 22 | 2380 | 1570 | 65.9 | 0.63 |
| 23 | 2000 | 1180 | 59.0 | 0.47 |
| 24 | 2380 | 1400 | 58.8 | 0.56 |
| 25 | 2095 | 1235 | 58.9 | 0.49 |
| 26 | 2000 | 1200 | 60.0 | 0.48 |
| 27 | 2000 | 1300 | 65.0 | 0.52 |
| 28 | 2095 | 1275 | 60.8 | 0.51 |
| 29 | 2190 | 1335 | 60.9 | 0.53 |
| 30 | 2095 | 1275 | 60.8 | 0.51 |
| 31 | 2190 | 1270 | 57.9 | 0.51 |
| 32 | 2285 | 1440 | 63.0 | 0.57 |
| 33 | 2190 | 1380 | 63.0 | 0.55 |
| 34 | 2000 | 1260 | 63.0 | 0.50 |
| 35 | 2000 | 1280 | 64.0 | 0.51 |
| 36 | 2190 | 1380 | 63.0 | 0.55 |
| 37 | 2095 | 1340 | 63.9 | 0.53 |
| 38 | 2190 | 1425 | 65.0 | 0.57 |
| 39 | 2095 | 1235 | 58.9 | 0.49 |
| 40 | 2095 | 1320 | 63.0 | 0.53 |
| 41 | 2095 | 1320 | 63.0 | 0.53 |
| 42 | 2000 | 1280 | 64.0 | 0.51 |
| 43 | 2000 | 1300 | 65.0 | 0.52 |
| 44 | 2000 | 1340 | 67.0 | 0.53 |
| 45 | 2095 | 1340 | 63.9 | 0.53 |
| 46 | 2000 | 1280 | 64.0 | 0.51 |
| 47 | 2000 | 1260 | 63.0 | 0.50 |
| 48 | 2095 | 1320 | 63.0 | 0.53 |
| 49 | 2000 | 1220 | 61.0 | 0.49 |
| 50 | 2095 | 1150 | 54.8 | 0.46 |
| 51 | 2095 | 1000 | 47.7 | 0.40 |
| 52 | 2000 | 1200 | 60.0 | 0.48 |
| 53 | 1905 | 1090 | 57.2 | 0.43 |
| 54 | 1905 | 1100 | 57.7 | 0.44 |
| 55 | 1620 | 1200 | 74.0 | 0.48 |
| 56 | 1905 | 1200 | 62.9 | 0.48 |
| 57 | 1620 | 1100 | 67.9 | 0.44 |
| 58 | 1930 | 1220 | 63.2 | 0.49 |
| 59 | 1905 | 1100 | 57.7 | 0.44 |
| 60 | 1905 | 1000 | 52.4 | 0.40 |
| **Coconut coir (15%)** | | | | |
| 1 | 2285 | 1415 | 61.9 | 0.57 |
| 2 | 1500 | 1080 | 72.0 | 0.43 |
| 3 | 1400 | 855 | 61.0 | 0.34 |
| 4 | 2500 | 1425 | 57.0 | 0.57 |
| 5 | 2300 | 1355 | 58.9 | 0.54 |
| 6 | 1810 | 1095 | 60.4 | 0.44 |
| 7 | 2000 | 1230 | 61.5 | 0.49 |
| 8 | 2285 | 1575 | 68.9 | 0.63 |
| 9 | 1715 | 1065 | 62.0 | 0.43 |
| 10 | 2285 | 1460 | 63.8 | 0.59 |
| 11 | 1810 | 1195 | 66.0 | 0.48 |
| 12 | 1905 | 1095 | 57.4 | 0.44 |
| 13 | 2190 | 1420 | 64.8 | 0.57 |
| 14 | 2380 | 1620 | 68.0 | 0.65 |
| 15 | 2570 | 1720 | 66.9 | 0.69 |
| 16 | 2190 | 1380 | 63.0 | 0.55 |
| 17 | 2570 | 1645 | 64.0 | 0.66 |
| 18 | 2000 | 1275 | 63.7 | 0.51 |
| 19 | 2380 | 1525 | 64.0 | 0.61 |
| 20 | 1715 | 1085 | 63.2 | 0.44 |
| 21 | 2190 | 1335 | 60.9 | 0.54 |
| 22 | 2570 | 1565 | 60.8 | 0.63 |
| 23 | 2380 | 1500 | 63.0 | 0.60 |
| 24 | 2570 | 2260 | 87.9 | 0.91 |
| 25 | 2380 | 1500 | 63.0 | 0.60 |
| 26 | 2380 | 1475 | 61.9 | 0.59 |
| 27 | 2475 | 1600 | 64.6 | 0.64 |
| 28 | 2285 | 1505 | 65.8 | 0.60 |
| 29 | 2380 | 1560 | 65.5 | 0.63 |
| 30 | 2380 | 1495 | 62.8 | 0.60 |
| 31 | 2570 | 1585 | 61.6 | 0.64 |
| 32 | 2285 | 1515 | 66.3 | 0.61 |
| 33 | 2140 | 1410 | 65.8 | 0.57 |
| 34 | 2380 | 1495 | 62.8 | 0.60 |
| 35 | 2380 | 1555 | 65.3 | 0.63 |
| 36 | 2285 | 1490 | 65.2 | 0.60 |
| 37 | 2190 | 1410 | 64.3 | 0.57 |
| 38 | 2285 | 1370 | 59.9 | 0.55 |
| 39 | 2285 | 1430 | 62.5 | 0.57 |
| 40 | 2285 | 1495 | 65.4 | 0.60 |
| 41 | 2095 | 1340 | 63.9 | 0.54 |
| 42 | 2285 | 1450 | 63.4 | 0.58 |
| 43 | 2475 | 1500 | 60.6 | 0.60 |
| 44 | 2475 | 1480 | 59.7 | 0.59 |
| 45 | 2475 | 1635 | 66.0 | 0.66 |
| 46 | 2380 | 1570 | 65.9 | 0.63 |
| 47 | 2285 | 1525 | 66.7 | 0.61 |
| 48 | 2285 | 1460 | 63.8 | 0.59 |
| 49 | 2475 | 1585 | 64.0 | 0.64 |
| 50 | 2475 | 1580 | 63.8 | 0.64 |
| 51 | 2285 | 1450 | 63.4 | 0.58 |
| 52 | 2285 | 1460 | 63.8 | 0.59 |
| 53 | 2190 | 1585 | 72.3 | 0.64 |
| 54 | 2285 | 1580 | 69.1 | 0.64 |
| 55 | 2095 | 1450 | 69.2 | 0.58 |
| 56 | 2190 | 1350 | 61.6 | 0.54 |
| 57 | 2190 | 1420 | 64.8 | 0.57 |
| 58 | 2095 | 1400 | 66.8 | 0.56 |
| 59 | 2095 | 1400 | 66.8 | 0.56 |
| 60 | 2190 | 1400 | 63.9 | 0.56 |
