# Supplementary material for: Bio-hydrogen production by co-digestion of domestic wastewater and biodiesel industry effluent
Source: PLoS One. 2018 Jul 11;13(7):e0199059. doi: 10.1371/journal.pone.0199059 (PMC6040696; doi:10.1371/journal.pone.0199059)
Supplement: S4 Table — (DOCX) [file pone.0199059.s004.docx]

**S4 Table: Effect of Effluent Recycling on continuous culture hydrogen production by *Bacillus amyloliquefaciens* CD16**

| **DAI** | **Biogas (mL)** | **Hydrogen** | | **Yield** |
| --- | --- | --- | --- | --- |
|  |  | **Volume (mL)** | **%** |  |
| **No effluent recycling** | | | | |
| 1 | 2475 | 1540 | 62.2 | 0.62 |
| 2 | 2570 | 1520 | 59.1 | 0.61 |
| 3 | 2475 | 1435 | 57.9 | 0.58 |
| 4 | 2380 | 1380 | 57.9 | 0.55 |
| 5 | 2380 | 1425 | 59.8 | 0.57 |
| 6 | 2380 | 1490 | 62.6 | 0.60 |
| 7 | 2285 | 1405 | 61.4 | 0.56 |
| 8 | 2285 | 1405 | 61.4 | 0.56 |
| 9 | 2285 | 1405 | 61.4 | 0.56 |
| 10 | 2380 | 1450 | 60.9 | 0.58 |
| 11 | 2475 | 1515 | 61.2 | 0.61 |
| 12 | 2475 | 1515 | 61.2 | 0.61 |
| 13 | 2380 | 1505 | 63.2 | 0.60 |
| 14 | 2380 | 1470 | 61.7 | 0.59 |
| 15 | 2380 | 1450 | 60.9 | 0.58 |
| 16 | 2475 | 1525 | 61.6 | 0.61 |
| 17 | 2380 | 1555 | 65.3 | 0.63 |
| 18 | 2285 | 1415 | 61.9 | 0.57 |
| 19 | 2285 | 1455 | 63.6 | 0.58 |
| 20 | 2285 | 1415 | 61.9 | 0.57 |
| 21 | 2190 | 1385 | 63.2 | 0.56 |
| 22 | 2285 | 1420 | 62.1 | 0.57 |
| 23 | 2285 | 1330 | 58.2 | 0.53 |
| 24 | 2285 | 1395 | 61.0 | 0.56 |
| 25 | 2285 | 1405 | 61.4 | 0.56 |
| 26 | 1430 | 870 | 60.8 | 0.35 |
| 27 | 1525 | 925 | 60.6 | 0.37 |
| 28 | 2095 | 1315 | 62.7 | 0.53 |
| 29 | 2285 | 1360 | 59.5 | 0.55 |
| 30 | 2380 | 1435 | 60.2 | 0.58 |
| 31 | 2380 | 1490 | 62.6 | 0.60 |
| 32 | 2285 | 1330 | 58.2 | 0.53 |
| 33 | 2285 | 1375 | 60.1 | 0.55 |
| 34 | 2285 | 1455 | 63.6 | 0.58 |
| 35 | 2285 | 1370 | 59.9 | 0.55 |
| 36 | 1810 | 1070 | 59.1 | 0.43 |
| 37 | 2380 | 1450 | 60.9 | 0.58 |
| 38 | 2475 | 1550 | 62.6 | 0.62 |
| 39 | 2380 | 1435 | 60.2 | 0.58 |
| 40 | 2285 | 1425 | 62.3 | 0.57 |
| 41 | 2000 | 1205 | 60.2 | 0.48 |
| 42 | 2095 | 1250 | 59.6 | 0.50 |
| 43 | 2000 | 1240 | 62.0 | 0.50 |
| 44 | 2095 | 1250 | 59.6 | 0.50 |
| 45 | 2190 | 1410 | 64.3 | 0.57 |
| 46 | 1905 | 1180 | 61.9 | 0.47 |
| 47 | 2000 | 1225 | 61.2 | 0.49 |
| 48 | 1905 | 1175 | 61.6 | 0.47 |
| 49 | 1905 | 1250 | 65.6 | 0.50 |
| 50 | 1810 | 1130 | 62.4 | 0.45 |
| 51 | 1810 | 1130 | 62.4 | 0.45 |
| 52 | 1905 | 1200 | 62.9 | 0.48 |
| 53 | 1810 | 1100 | 60.7 | 0.44 |
| 54 | 1905 | 1220 | 64.0 | 0.49 |
| 55 | 1905 | 1250 | 65.6 | 0.50 |
| 56 | 1905 | 1240 | 65.0 | 0.50 |
| 57 | 1810 | 1140 | 62.9 | 0.46 |
| 58 | 1905 | 1230 | 64.5 | 0.49 |
| 59 | 1905 | 1230 | 64.5 | 0.49 |
| 60 | 1905 | 1240 | 65.0 | 0.50 |
| **25% effluent recycling** | | | | |
| 1 | 2475 | 1435 | 57.9 | 0.58 |
| 2 | 2570 | 1480 | 57.5 | 0.59 |
| 3 | 2665 | 1625 | 60.9 | 0.65 |
| 4 | 2570 | 1515 | 58.9 | 0.61 |
| 5 | 2665 | 1520 | 57.0 | 0.61 |
| 6 | 2665 | 1490 | 55.9 | 0.60 |
| 7 | 2570 | 1415 | 55.0 | 0.57 |
| 8 | 2570 | 1415 | 55.0 | 0.57 |
| 9 | 2285 | 1315 | 57.5 | 0.53 |
| 10 | 2380 | 1375 | 57.7 | 0.55 |
| 11 | 2380 | 1405 | 59.0 | 0.56 |
| 12 | 2285 | 1300 | 56.8 | 0.52 |
| 13 | 2285 | 1370 | 59.9 | 0.55 |
| 14 | 2190 | 1250 | 57.0 | 0.50 |
| 15 | 2190 | 1210 | 55.2 | 0.49 |
| 16 | 2190 | 1305 | 59.5 | 0.52 |
| 17 | 2095 | 1205 | 57.5 | 0.48 |
| 18 | 2000 | 1150 | 57.5 | 0.46 |
| 19 | 2285 | 1215 | 53.1 | 0.49 |
| 20 | 2190 | 1255 | 57.3 | 0.50 |
| 21 | 2095 | 1190 | 56.8 | 0.48 |
| 22 | 2095 | 1190 | 56.8 | 0.48 |
| 23 | 2190 | 1290 | 58.9 | 0.52 |
| 24 | 2095 | 1235 | 58.9 | 0.50 |
| 25 | 2190 | 1295 | 59.1 | 0.52 |
| 26 | 2095 | 1220 | 58.2 | 0.49 |
| 27 | 1430 | 845 | 59.0 | 0.34 |
| 28 | 950 | 560 | 58.9 | 0.23 |
| 29 | 1905 | 1110 | 58.2 | 0.45 |
| 30 | 2000 | 1145 | 57.2 | 0.46 |
| 31 | 2095 | 1235 | 58.9 | 0.50 |
| 32 | 2000 | 1190 | 59.5 | 0.48 |
| 33 | 1905 | 1100 | 57.7 | 0.44 |
| 34 | 1810 | 1050 | 58.0 | 0.42 |
| 35 | 1620 | 1080 | 66.6 | 0.43 |
| 36 | 1525 | 880 | 57.7 | 0.35 |
| 37 | 1525 | 885 | 58.0 | 0.36 |
| 38 | 1525 | 900 | 59.0 | 0.36 |
| 39 | 1620 | 990 | 61.1 | 0.40 |
| 40 | 1620 | 960 | 59.2 | 0.39 |
| 41 | 1620 | 900 | 55.5 | 0.36 |
| 42 | 1715 | 1035 | 60.3 | 0.42 |
| 43 | 1810 | 1065 | 58.8 | 0.43 |
| 44 | 1715 | 990 | 57.7 | 0.40 |
| 45 | 1525 | 900 | 59.0 | 0.36 |
| 46 | 1620 | 945 | 58.3 | 0.38 |
| 47 | 1620 | 945 | 58.3 | 0.38 |
| 48 | 1620 | 1000 | 61.7 | 0.40 |
| 49 | 1620 | 1040 | 64.1 | 0.42 |
| 50 | 1715 | 1000 | 58.3 | 0.40 |
| 51 | 1715 | 1020 | 59.4 | 0.41 |
| 52 | 1620 | 1000 | 61.7 | 0.40 |
| 53 | 1620 | 1030 | 63.5 | 0.41 |
| 54 | 1620 | 1040 | 64.1 | 0.42 |
| 55 | 1525 | 1000 | 65.5 | 0.40 |
| 56 | 1525 | 1000 | 65.5 | 0.40 |
| 57 | 1620 | 1020 | 62.9 | 0.41 |
| 58 | 1620 | 1000 | 61.7 | 0.40 |
| 59 | 1620 | 1020 | 62.9 | 0.41 |
| 60 | 1620 | 1030 | 63.5 | 0.41 |
| **50% effluent recycling** | | | | |
| 1 | 2380 | 1215 | 51.0 | 0.49 |
| 2 | 2285 | 1235 | 54.0 | 0.50 |
| 3 | 2380 | 1285 | 53.9 | 0.52 |
| 4 | 2380 | 1285 | 53.9 | 0.52 |
| 5 | 2380 | 1330 | 55.8 | 0.53 |
| 6 | 2000 | 1100 | 55.0 | 0.44 |
| 7 | 2285 | 1280 | 56.0 | 0.51 |
| 8 | 1715 | 945 | 55.1 | 0.38 |
| 9 | 2000 | 1100 | 55.0 | 0.44 |
| 10 | 1905 | 1065 | 55.9 | 0.43 |
| 11 | 1810 | 1015 | 56.0 | 0.41 |
| 12 | 1905 | 1050 | 55.1 | 0.42 |
| 13 | 2000 | 1140 | 57.0 | 0.46 |
| 14 | 1905 | 1030 | 54.0 | 0.41 |
| 15 | 1810 | 1015 | 56.0 | 0.41 |
| 16 | 1430 | 770 | 53.8 | 0.31 |
| 17 | 1610 | 870 | 54.0 | 0.35 |
| 18 | 1525 | 950 | 62.2 | 0.38 |
| 19 | 1430 | 845 | 59.0 | 0.34 |
| 20 | 1240 | 695 | 56.0 | 0.28 |
| 21 | 950 | 520 | 54.7 | 0.21 |
| 22 | 860 | 430 | 50.0 | 0.17 |
| 23 | 1140 | 580 | 50.8 | 0.23 |
| 24 | 950 | 580 | 61.0 | 0.23 |
| 25 | 570 | 300 | 52.6 | 0.12 |
| 26 | 665 | 405 | 60.9 | 0.16 |
| 27 | 760 | 395 | 51.9 | 0.16 |
| 28 | 665 | 360 | 54.1 | 0.14 |
| 29 | 475 | 200 | 42.1 | 0.08 |
| 30 | 285 | 150 | 52.6 | 0.06 |
| 31 | 200 | 150 | 75.0 | 0.06 |
| 32 | 150 | 90 | 60.0 | 0.04 |
| 33 | 150 | 100 | 66.6 | 0.04 |
| 34 | 120 | 50 | 41.6 | 0.02 |
| 35 | 100 | 45 | 45.0 | 0.02 |
| 36 | 100 | 50 | 50.0 | 0.02 |
| 37 | 90 | 50 | 55.5 | 0.02 |
| 38 | 0 | 0 | N.A | N.A |
| 39 | 0 | 0 | N.A | N.A |
| 40 | 0 | 0 | N.A | N.A |
| 41 | 0 | 0 | N.A | N.A |
| 42 | 0 | 0 | N.A | N.A |
| 43 | 0 | 0 | N.A | N.A |
| 44 | 0 | 0 | N.A | N.A |
| 45 | 0 | 0 | N.A | N.A |
| 46 | 0 | 0 | N.A | N.A |
| 47 | 0 | 0 | N.A | N.A |
| 48 | 0 | 0 | N.A | N.A |
| 49 | 0 | 0 | N.A | N.A |
| 50 | 0 | 0 | N.A | N.A |
| 51 | 0 | 0 | N.A | N.A |
| 52 | 0 | 0 | N.A | N.A |
| 53 | 0 | 0 | N.A | N.A |
| 54 | 0 | 0 | N.A | N.A |
| 55 | 0 | 0 | N.A | N.A |
| 56 | 0 | 0 | N.A | N.A |
| 57 | 0 | 0 | N.A | N.A |
| 58 | 0 | 0 | N.A | N.A |
| 59 | 0 | 0 | N.A | N.A |
| 60 | 0 | 0 | N.A | N.A |
| **75% effluent recycling** | | | | |
| 1 | 2285 | 1285 | 56.2 | 0.52 |
| 2 | 1620 | 940 | 58.0 | 0.38 |
| 3 | 1715 | 965 | 56.2 | 0.39 |
| 4 | 1715 | 965 | 56.2 | 0.39 |
| 5 | 1430 | 800 | 55.9 | 0.32 |
| 6 | 1525 | 800 | 52.4 | 0.32 |
| 7 | 1525 | 760 | 49.8 | 0.31 |
| 8 | 1430 | 740 | 51.7 | 0.30 |
| 9 | 1240 | 620 | 50.0 | 0.25 |
| 10 | 950 | 495 | 52.1 | 0.20 |
| 11 | 950 | 520 | 54.7 | 0.21 |
| 12 | 950 | 495 | 52.1 | 0.20 |
| 13 | 950 | 475 | 50.0 | 0.19 |
| 14 | 665 | 330 | 49.6 | 0.13 |
| 15 | 760 | 395 | 51.9 | 0.16 |
| 16 | 950 | 515 | 54.2 | 0.21 |
| 17 | 570 | 290 | 50.8 | 0.12 |
| 18 | 475 | 250 | 52.6 | 0.10 |
| 19 | 380 | 195 | 51.3 | 0.08 |
| 20 | 380 | 215 | 56.5 | 0.09 |
| 21 | 380 | 225 | 59.2 | 0.09 |
| 22 | 475 | 245 | 51.5 | 0.10 |
| 23 | 380 | 190 | 50.0 | 0.08 |
| 24 | 285 | 160 | 56.1 | 0.06 |
| 25 | 285 | 160 | 56.1 | 0.06 |
| 26 | 190 | 100 | 52.6 | 0.04 |
| 27 | 190 | 95 | 50.0 | 0.04 |
| 28 | 95 | 45 | 47.3 | 0.02 |
| 29 | 95 | 50 | 52.6 | 0.02 |
| 30 | 95 | 40 | 42.1 | 0.02 |
| 31 | 80 | 50 | 62.5 | 0.02 |
| 32 | 80 | 50 | 62.5 | 0.02 |
| 33 | 85 | 40 | 47.0 | 0.02 |
| 34 | 50 | 20 | 40.0 | 0.01 |
| 35 | 50 | 20 | 40.0 | 0.01 |
| 36 | 50 | 20 | 40.0 | 0.01 |
| 37 | 0 | 0 | N.A | N.A |
| 38 | 0 | 0 | N.A | N.A |
| 39 | 0 | 0 | N.A | N.A |
| 40 | 0 | 0 | N.A | N.A |
| 41 | 0 | 0 | N.A | N.A |
| 42 | 0 | 0 | N.A | N.A |
| 43 | 0 | 0 | N.A | N.A |
| 44 | 0 | 0 | N.A | N.A |
| 45 | 0 | 0 | N.A | N.A |
| 46 | 0 | 0 | N.A | N.A |
| 47 | 0 | 0 | N.A | N.A |
| 48 | 0 | 0 | N.A | N.A |
| 49 | 0 | 0 | N.A | N.A |
| 50 | 0 | 0 | N.A | N.A |
| 51 | 0 | 0 | N.A | N.A |
| 52 | 0 | 0 | N.A | N.A |
| 53 | 0 | 0 | N.A | N.A |
| 54 | 0 | 0 | N.A | N.A |
| 55 | 0 | 0 | N.A | N.A |
| 56 | 0 | 0 | N.A | N.A |
| 57 | 0 | 0 | N.A | N.A |
| 58 | 0 | 0 | N.A | N.A |
| 59 | 0 | 0 | N.A | N.A |
| 60 | 0 | 0 | N.A | N.A |
